# Effects of plant growth-promoting rhizobacteria on co-inoculation with *Bradyrhizobium* in soybean crop: a meta-analysis of studies from 1987 to 2018

**DOI:** 10.7717/peerj.7905

**Published:** 2020-01-09

**Authors:** Douglas M. Zeffa, Lucas H. Fantin, Alessandra Koltun, André L.M. de Oliveira, Maria P.B.A. Nunes, Marcelo G. Canteri, Leandro S.A. Gonçalves

**Affiliations:** 1 Department of Agronomy, Universidade Estadual de Maringá, Maringá, Paraná, Brazil; 2 Department of Agronomy, Universidade Estadual de Londrina, Londrina, Paraná, Brazil; 3 Department of Biochemistry and Biotechnology, Universidade Estadual de Londrina, Londrina, Paraná, Brazil

**Keywords:** *Glycine max* (L.) Merrill, Bacteria, Agriculture, Biofertilizer, Plant productivity

## Abstract

**Background:**

The co-inoculation of soybean with *Bradyrhizobium* and other plant growth-promoting rhizobacteria (PGPR) is considered a promising technology. However, there has been little quantitative analysis of the effects of this technique on yield variables. In this context, the present study aiming to provide a quantification of the effects of the co-inoculation of *Bradyrhizobium* and PGPR on the soybean crop using a meta-analysis approach.

**Methods:**

A total of 42 published articles were examined, all of which considered the effects of co-inoculation of PGPR and *Bradyrhizobium* on the number of nodules, nodule biomass, root biomass, shoot biomass, shoot nitrogen content, and grain yield of soybean. We also determined whether the genus of the PGPR used as co-inoculant, as well as the experimental conditions, altered the effect size of the PGPR.

**Results:**

The co-inoculation technology resulted in a significant increase in nodule number (11.40%), nodule biomass (6.47%), root biomass (12.84%), and shoot biomass (6.53%). Despite these positive results, no significant increase was observed in shoot nitrogen content and grain yield. The response of the co-inoculation varied according to the PGPR genus used as co-inoculant, as well as with the experimental conditions. In general, the genera *Azospirillum*, *Bacillus*, and *Pseudomonas* were more effective than *Serratia*. Overall, the observed increments were more pronounced under pot than that of field conditions. Collectively, this study summarize that co-inoculation improves plant development and increases nodulation, which may be important in overcoming nutritional limitations and potential stresses during the plant growth cycle, even though significant increases in grain yield have not been evidenced by this data meta-analysis.

## Introduction

The soybean crop (*Glycine max* (L.) Merrill) is one of the main commodities in the world, mainly for its high protein and oil contents, favoring its use in several areas of the agroindustry (Hart, 2017; Nguyen, 2018). In countries such as Brazil and Argentina, some of the world’s leading producers, soybean is a highly profitable crop for farmers, since its nitrogen (N) requirements are fully met by biological nitrogen fixation (BNF) (Hungria et al., 2005). In BNF, the soybean establishes a symbiotic relationship with rhizobia, providing photoassimilates in exchange for biologically active N (Hungria, Menna & Delamuta, 2015; Gresshoff, 2018). These microorganisms usually inhabit the plant root system, where they colonize and grow endophytically, producing the enzyme complex nitrogenase, which allows them to convert atmospheric nitrogen (N_2_) to ammonia and its further incorporation into biomolecules in several forms of organic N (Hungria et al., 2006; Oldroyd, 2013; Hungria, Nogueira & Araujo, 2013).

The genus *Bradyrhizobium* (Jordan, 1982) is considered the main rhizobial genus that establishes a symbiotic association with soybean (Hungria, Nogueira & Araujo, 2015; Sugiyama et al., 2015; Schmidt, Messmer & Wilbois, 2015). According to List of Prokaryotic Names with Standing in Nomenclature (LPSN, 2019), 41 species of *Bradyrhizobium* have already been described, with the species *B. elkanii*, *B. japonicum*, and *B. diazoefficiens* being the most used in commercial inoculants (Siqueira et al., 2014; Schmidt, Messmer & Wilbois, 2015; Delamuta et al., 2017). The *Bradyrhizobium*-soybean symbiosis is considered one of the most important natural relations exploited by the agricultural activity, since these bacteria can lead to grain yield increase and, consequently, eliminate or reduce the dependence on inorganic N fertilizers in crop cultivation (Chang, Lee & Hungria, 2015; Hungria, Marco & Ricardo, 2015; Collino et al., 2015).

In addition to the use of rhizobia, another strategy that has been employed to increase soybean productivity is the co-inoculation of *Bradyrhizobium* with other genera of plant growth-promoting rhizobacteria (PGPR), such as *Azospirillum* (Hungria, Marco & Ricardo, 2015; Zuffo et al., 2016), *Bacillus* (Mishra et al., 2009; Tonelli, Magallanes-Noguera & Fabra, 2017), *Pseudomonas* (Egamberdieva, Jabborova & Berg, 2016; Pawar et al., 2018), and *Serratia* (Bai, 2002; Pan, Vessey & Smith, 2002). These microorganisms act as promoters of plant growth via the production of amino acids, indole acetic acid (IAA), gibberellins, and other polyamines, improving root growth and, consequently, increasing water and nutrient absorption by the plants and generating rhizobia-soybean interaction sites (Schmidt, Messmer & Wilbois, 2015; Yadav et al., 2017). Among other benefits, PGPR are also able to solubilize phosphates, produce siderophores, fix N_2_, and mitigate biotic and abiotic stresses (Ahemad & Kibret, 2014; Olanrewaju, Glick & Babalola, 2017). In the sense, the co-inoculation of microorganisms with different functions can be considered an economically viable and environmentally sustainable strategy to improve plant performance (Muthukumar & Udaiyan, 2018; Yan, Zhu & Yang, 2018).

Although it is considered a promising technology, the co-inoculation of soybean has shown contrasting results (Schmidt, Messmer & Wilbois, 2015). Hungria, Nogueira & Araujo (2013) investigating the effects of co-inoculation of soybean seeds with *B. japonicum* and *A. brasilense*, observed an average increase of 420 kg ha^−1^ (16.1%) compared to the control treatment inoculated only with *B. japonicum*. Conversely, Zuffo et al. (2016) reported no significant differences in grain yield between inoculated (*B. japonicum*) and co-inoculated (*B. japonicum* + *A. brasilense*) treatments for six soybean cultivars. Nevertheless, Atieno et al. (2012) observed that co-inoculation of *B. japonicum* and *B. subtilis* increased traits related to soybean nodulation and biomass. Therefore, what is not yet clear is the impact of co-inoculation on soybean grain yield. In view of this, the statistical technique known as meta-analysis may be a powerful tool to determine the real effects of the co-inoculation of PGPR and *Bradyrhizobium* on soybean cultivation, since this technique allows the quantitative combination of results from different studies, leading to a synthesis of results with high power and reliability. Therefore, the objective of this study was to investigate and solve the inconsistency of results using a meta-analysis.

## Material & Methods

### Bibliographic research and data collection

**Figure 1:**
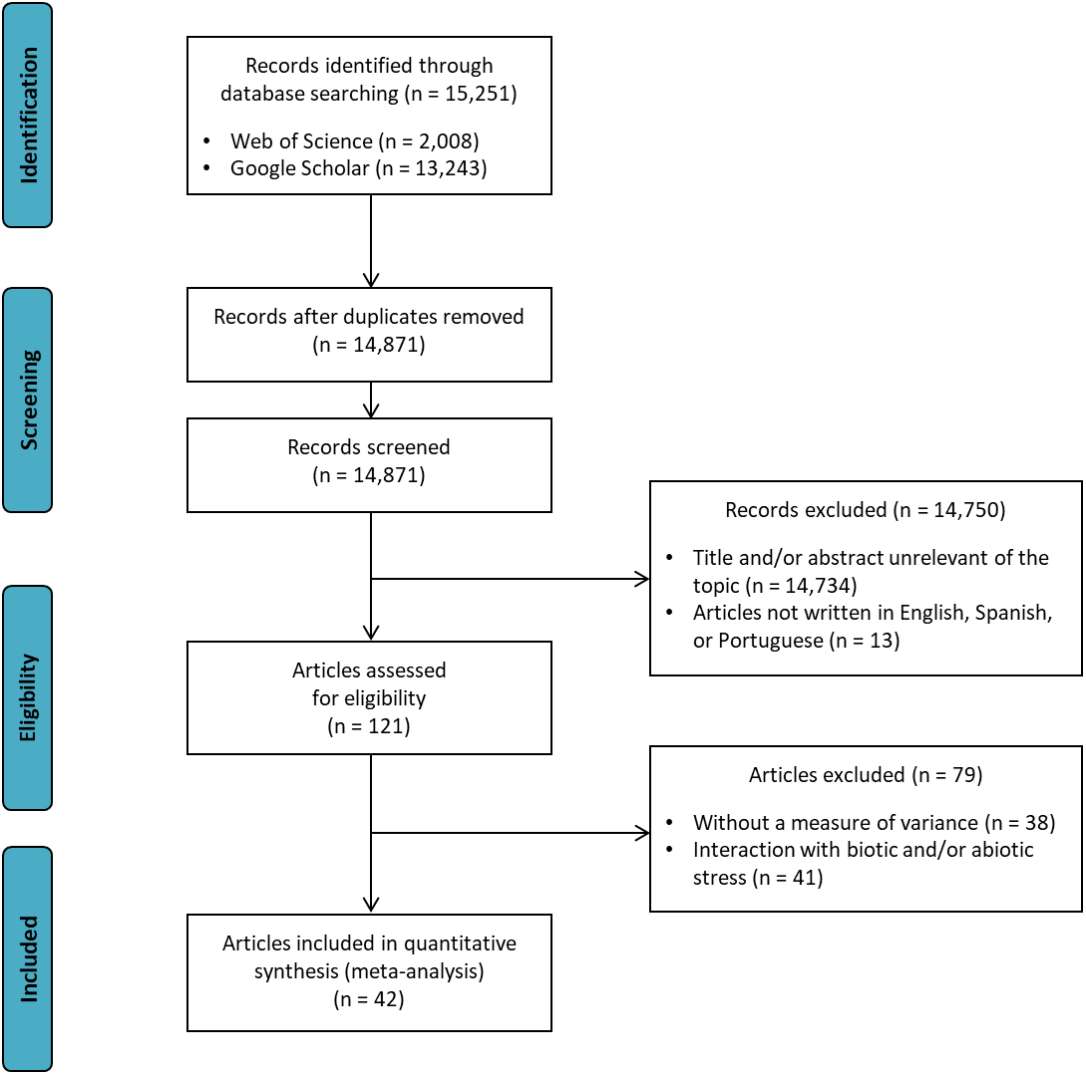
Preferred reporting items for systematic reviews and meta-analyses (PRISMA) flow diagram for the meta-analysis.

Figure 1 shows the search strategy for the review presented according to the PRISMA reporting guidelines (Liberati et al., 2009). Data were collected from articles published in scientific journals, which were obtained by a systematic literature review using the Web of Science® and Google Scholar® databases. The search strategy “soybean AND (co-inoculation OR PGPR)” was applied in both databases in February 2018 by two independent reviewers (DMZ and LHF). Discussion between the two reviewers resolved any differences. If no consensus could be reached, another reviewer (LSAG) resolved the conflict. After screening relevant titles and filtering out duplicates, 79 articles were reviewed. The final article number was then reduced to 42 based on the following criteria: (i) articles written in English, Spanish, or Portuguese; (ii) studies that presented a measure of variance: coefficient of variation (CV), mean square residual (MSR), standard error of the mean (SE), or standard deviation of the mean (SD); (iii) studies showing the number of nodules, nodule biomass, shoot biomass, root biomass, shoot N content, and/or grain yield traits; and (iv) studies comparing inoculated treatments (*Bradyrhizobium*) × co-inoculated (*Bradyrhizobium* + PGPR). Interaction data with biotic or abiotic stresses were not extracted from articles.

Nodule, root, and shoot biomass were generally presented as dry biomass; however, in some cases, the values of fresh biomass were used when they were the only type of measure available. For the variable shoot N content, protein content was also used as an indirect source. The means and the measures of variance were extracted from the article tables, when provided. For figures, we extracted data using the ImageJ 1.5 software (Pérez & Pascau, 2013). Bar graphs that contained variance without specification were considered as SD.

### Effect size and moderator variables

Estimates of the effects of the PGPR on the evaluated traits were obtained using the natural logarithmic response ratio (ln *R*) as effect size (Hedges, Gurevitch & Curtis, 1999):

\begin{eqnarray*}\ln \nolimits R=\ln \nolimits \left( \frac{Ti}{Tc} \right) \end{eqnarray*}

in which *Ti* is the mean of the co-inoculated treatment (*Bradyrhizobium* + PGPR) and *Tc* is the mean of the control treatment (*Bradyrhizobium*). The rate of the response is useful when different units are reported in the studies, while logarithmic transformation is necessary to properly balance the treatments of positive and negative effects to maintain symmetry within the analysis (Cooper, Hedges & Valentine, 2009). Thus, values above zero indicate an increase in the variable induced by PGPR, while values below zero reflect a reduction, and a value that equals zero means absence of the effect of PGPR. In addition, the ln *R* can be easily transformed into a percentage response (%R), using the following formula:

\begin{eqnarray*}\text{%}R=100\times [\exp \nolimits .(\ln \nolimits R)-1] \end{eqnarray*}

Experimental conditions (field or pot) and PGPR genera used in co-inoculation were used as moderator variables in the present study, since they may influence the response of soybean to the effects of co-inoculation. Moderator variables were selected based on the criterion of a minimum of 15 observations in at least two scientific articles. The moderator variables were tested even when the evaluated trait presented no significant value, since the positive results may have been diluted in the general effect.

### Meta-analysis

Prior to the construction of the meta-analysis models, data heterogeneity was verified by the *Q* (Cochran, 1954) and *I*^2^ (Higgins & Thompson, 2002) tests to determine the use of fixed or random/mixed-effects model approaches. The synthesis produced by the meta-analysis is balanced according to the weight of each of the studies, so that they can contribute individually to the meta-analytic result. In this study, the inverse variance method (Hedges, Gurevitch & Curtis, 1999) was used to assign the weights:

\begin{eqnarray*}Wi= \frac{1}{Vi} \end{eqnarray*}

in which *Wi* represents the weight assigned to the *i*-th study and *Vi* is the variance of the *i*-th study. Thus, the lower the study variance, the greater its contribution to the synthesis generated.

The estimates produced by the meta-analysis and their respective 95% confidence intervals (95% CI) were presented in forest plot graphs. Therefore, the mean effect size was considered significant when its 95% CI did not overlap with zero. Statistical analyses were performed in the software R (https://r-project.org), using the meta (Schwarzer, 2007), metafor (Viechtbauer, 2010), and ggplot2 (Wickham, 2016) packages.

## Results

### Metadata

**Figure 2:**
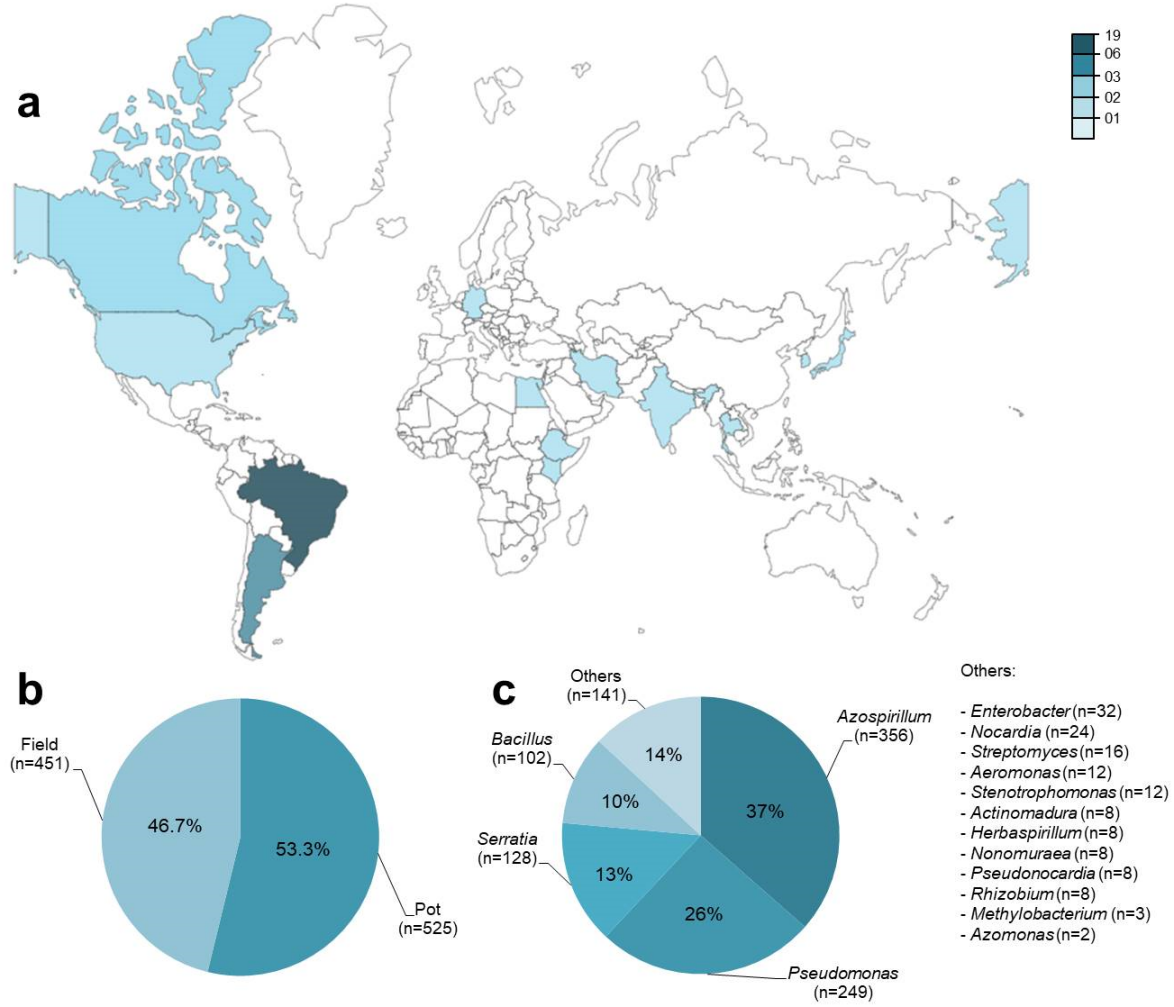
General data information (*n* = 976) obtained from 42 studies used in the meta-analysis, according to (A) location of the experiments, (B) experimental conditions and (C) genera of PGPR used as co-inoculants.

Metadata was obtained from 42 published articles from 13 countries between 1987 and 2018 (Fig. 2A; Table S1). A total of 976 observations (*n*) were obtained from an aggregate of 74 trials, where each observation included a co-inoculated treatment (PGPR + *Bradyrhizobium*) and a control treatment (*Bradyrhizobium*) for the number of nodules (*n* = 278), nodule biomass (*n* = 228), shoot N content (*n* = 88), and grain yield (*n* = 78). Among the observations, 53% (*n* = 525) were obtained in pots and 47% (*n* = 451) under field conditions (Fig. 2B). Except for grain yield, reported only under field conditions, all other traits were observed under pot and field conditions. A total of 16 different genera of PGPR were used as co-inoculants (Fig. 2C).

Heterogeneity on the full dataset was highly significant by the Cochran test (*Q* = 29822.77, *df* = 975, *p* < 0.0001). The *I*^2^ statistic also indicated high heterogeneity, which showed a magnitude of 96.40%. Due to the great heterogeneity of the observations, the meta-analysis was performed using random-effects models. Likewise, significant heterogeneity (*p* < 0.0001) was observed for the six evaluated traits grouped by the moderator variables, suggesting the use of mixed-effects models, in which we evaluated the moderator variables as random effect covariates and the observations as fixed effects (Cooper, Hedges & Valentine, 2009).

### General effect of co-inoculation

**Figure 3:**
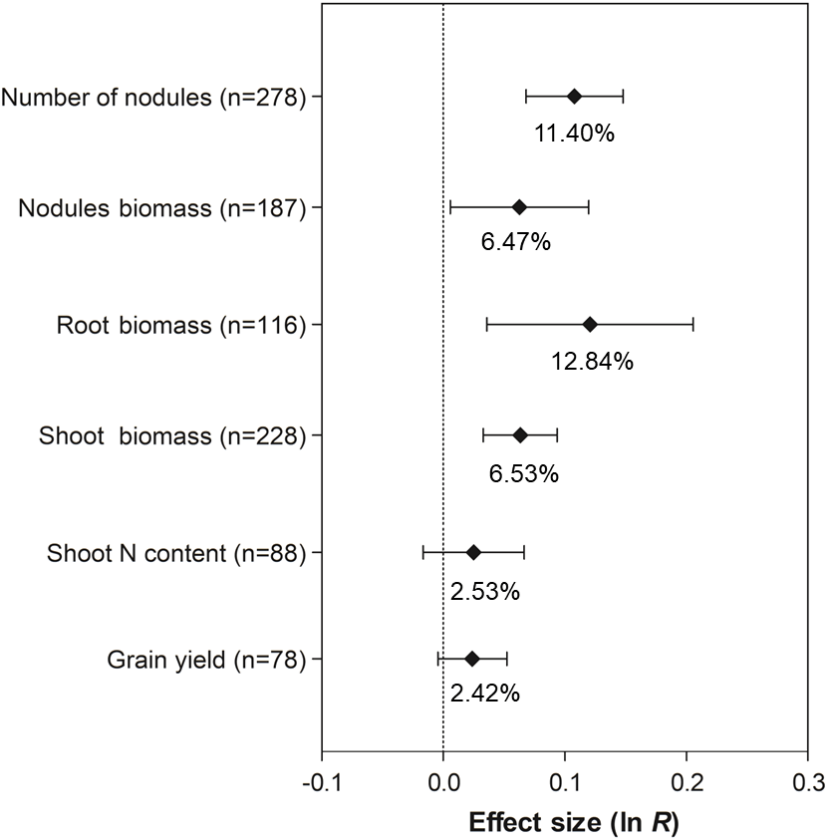
Effect sizes (ln *R*) of PGPR co-inoculation on nodule numbers, nodule biomass, root biomass, shoot biomass, shoot N content and grain yield. The graph reflects the parameter estimates from the random-effects meta-analysis model conducted for each variable, and the error bars represent the 95% confidence interval. The values below the effect size of each variable are the percentages of the PGPR effect (ln *R* transformed back to the original values).

The co-inoculation of soybean with PGPR showed a positive and significant effect on the number of nodules (11.40%, 95% CI [7.06 –15.93%]), nodule biomass (6.47%, 95% CI [0.59–12.70%]), root biomass (12.84%, 95% CI [3.64–22.85%]), and shoot biomass (6.53%, 95% CI [3.34–9.82%]) (Fig. 3). However, there was no increase in grain yield and shoot N content associated with co-inoculation, since their 95% CI overlapped with zero.

### Effects of the moderator variables

**Figure 4:**
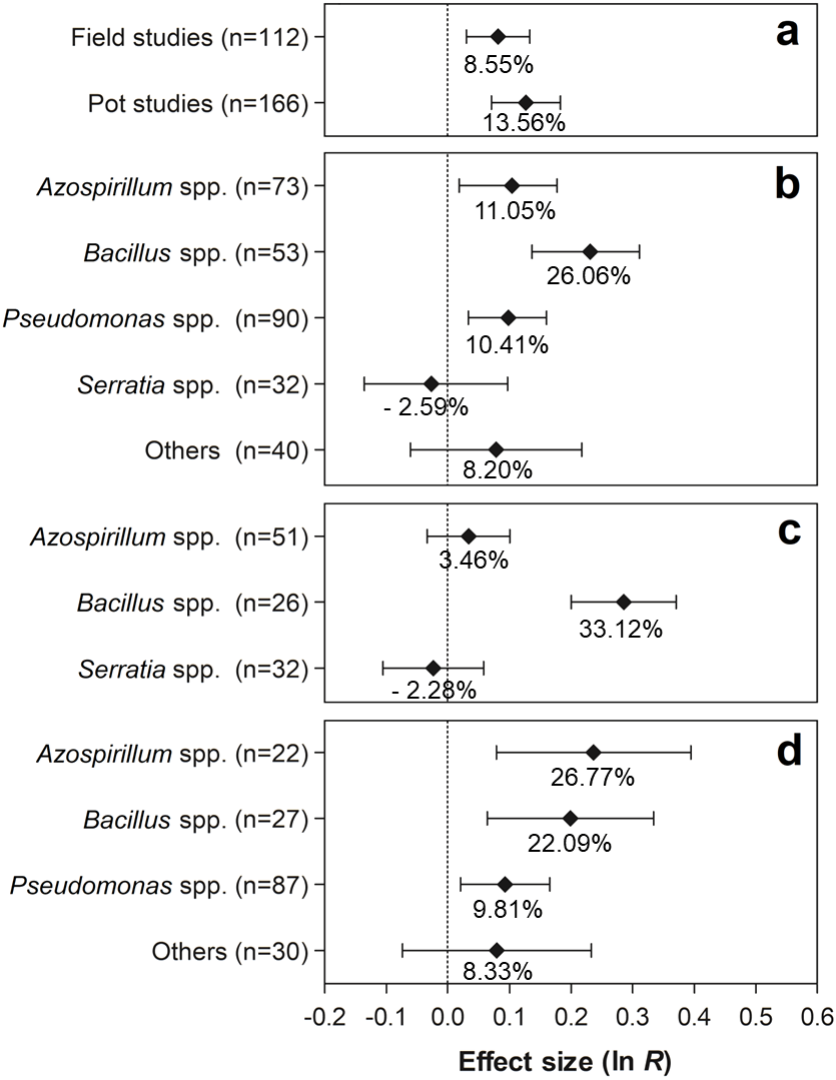
Effect sizes (ln *R*) of PGPR co-inoculation on number of nodules grouped by the moderator variables: (A) experimental conditions; (B) genera of PGPR; (C) genera of PGPR under field conditions; and (D) genera of PGPR under pot conditions. The graph reflects the parameter estimates from the random-effects meta-analysis model conducted for each variable, and the error bars represent the 95% confidence interval. The values below the effect size of each variable are the percentages of the PGPR effect (ln *R* transformed back to the original values).

The effects of the moderator variables on the number of nodules are shown in Fig. 4. Regarding the experimental conditions, the tests conducted under field and pot conditions showed significant effects of 8.55% (95% CI [3.09–14.29%]) and 12.84% (95% CI [7.38–20.12%]), respectively, on the evaluated traits (Fig. 4A). Both effect sizes can be considered similar, since the 95% CI overlapped considerably. Regarding the PGPR, the genera *Azospirillum*, *Bacillus*, and *Pseudomonas* showed positive effects for this moderator variable, increasing the number of nodules in 11.05% (95% CI [1.90–19.48%]), 26.05% (95% CI [14.71–36.59%]), and 10.41% (95% CI [3.43–17.41]), respectively (Fig. 4B). In relation to PGPR, only the genus *Bacillus* presented significant effects, leading to average increments of 33.12% (95% CI [22.27–44.93%]) (Fig. 4C). In contrast, in the pot experiments, the genera *Azospirillum*, *Bacillus*, and *Pseudomonas* presented significant effects of 26.77% (95% CI [8.26–48.44]), 22.09% (95% CI [6.67–39.72%]), and 9.81% (95% CI [2.13–26.30%]) on the number of nodules, respectively (Fig. 4D).

**Figure 5:**
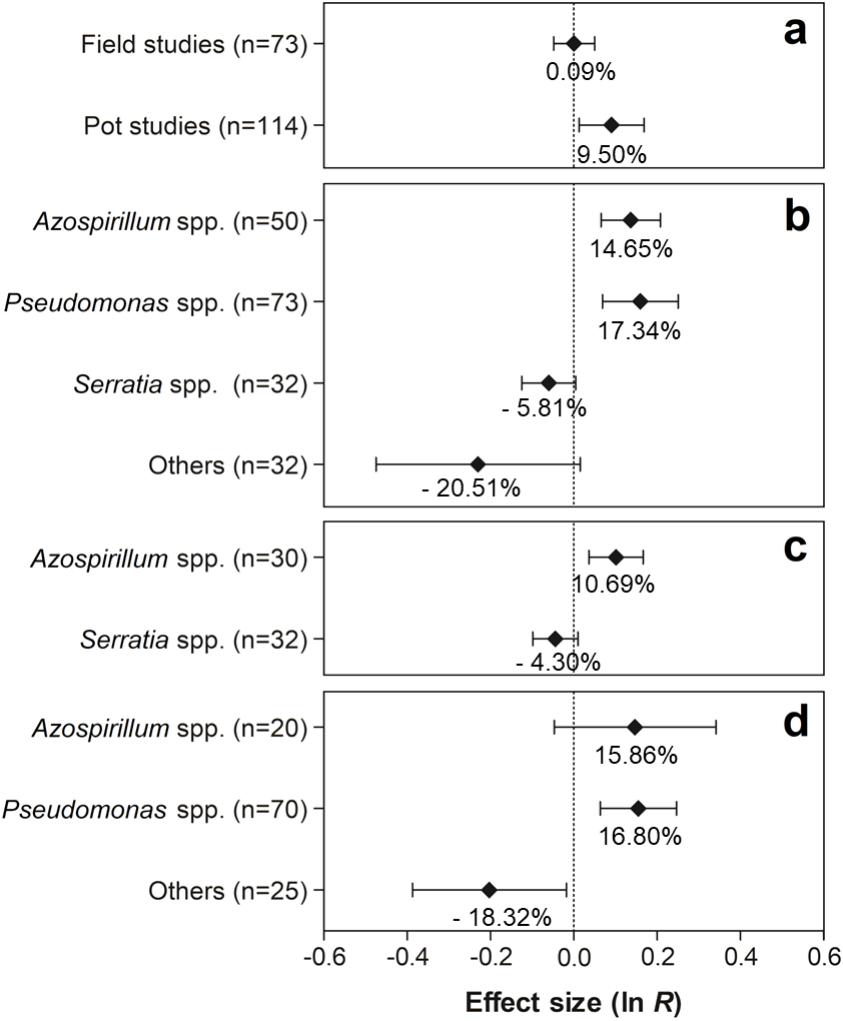
Effect sizes (ln *R*) of PGPR co-inoculation on nodule biomass grouped by the moderator variables: (A) experimental conditions; (B) genera of PGPR; (C) genera of PGPR under field conditions; and (D) genera of PGPR under pot conditions. The graph reflects the parameter estimates from the random-effects meta-analysis model and the error bars represents the 95% confidence interval. The values below the effect size of each variable are the percentages of the PGPR effect (ln *R* transformed back to the original values).

As shown in Fig. 5A, only the experiments conducted in pots showed significant effects on nodule biomass, with an average increase of 9.50% (95% CI [1.40–18.40%]). As for PGPR, the genera *Azospirillum* and *Pseudomonas* presented positive effects on this trait, showing increases of 14.65% (95% CI [6.76–23.13%]) and 17.34% (95% CI [7.17–29.49]), respectively (Fig. 5B). Although no significant effect of co-inoculation on nodule biomass was observed in the experiments conducted under field conditions, the partitioning of this effect in relation to the PGPR genera indicated a positive and significant effect of the genus *Azospirillum*, increasing the value of the trait in 10.69% (95% CI [3.70–18.16]) (Fig. 5C). In contrast, different PGPR in the pot studies revealed that only the genus *Pseudomonas* showed significant improvements in nodule biomass, presenting an increase of 16.80% (95% CI [6.58–27.90]) (Fig. 5D). On the other hand, a reduction of −18.32% in the average nodule biomass (95% CI [−32.08–1.74]) was observed by co-inoculation of other PGPR genera (*Actinomadura*, *Aeromonas*, *Bacillus*, *Enterobacter*, *Herbaspirillum*, *Nocardia*, *Nonomuraea*, *Pseudonocardia*, *Rhizobium*, and *Streptomyces*).

**Figure 6:**
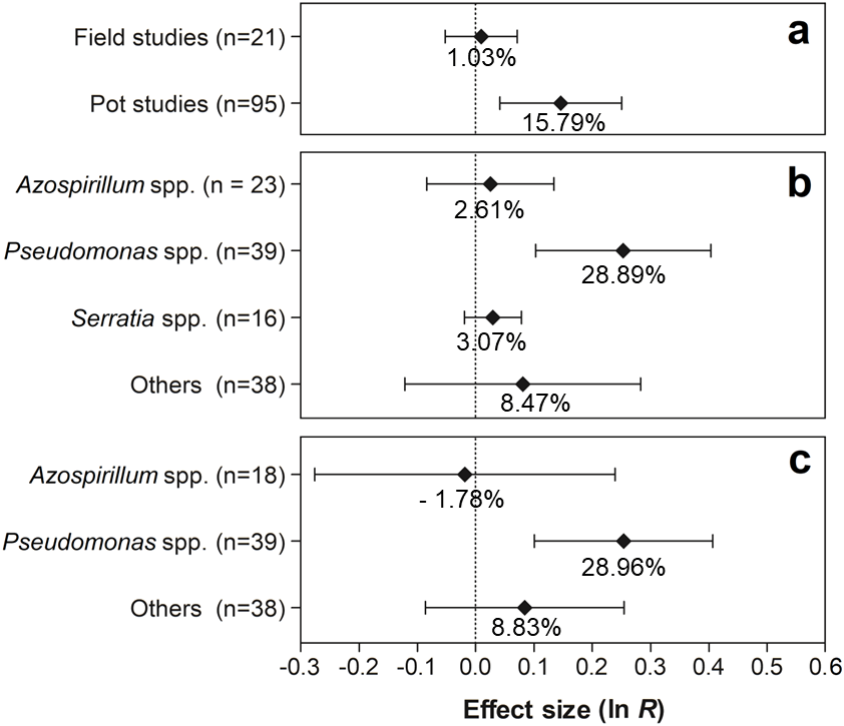
Effect sizes (ln *R*) of PGPR co-inoculation on root biomass grouped by the moderator variables: (A) experimental conditions; (B) genera of PGPR; and (C) genera of PGPR under pot conditions. The graph reflects the estimates of the effects of the parameter estimates from the random-effects meta-analysis model and the error bars represent the 95% confidence interval. The values below the effect size of each variable are the percentages of the PGPR effect (ln *R* transformed back to the original values).

The effects of the moderator variables on root biomass are presented in Fig. 6. For the experimental conditions, only the experiments conducted in pots showed significant values, with an increase of 15.79% (95% CI [4.33–28.49%]) in root biomass (Fig. 6A). Regarding PGPR, the genus *Pseudomonas* was the only one with a positive effect on this trait, presenting an increment of 28.89% (95% CI [10.93–49.77%]) (Fig. 6B). Furthermore, according to the results, only the genus *Pseudomonas* resulted in a significantly increased root biomass (28.96%) (95% CI [10.68–50.25%]) (Fig. 6C).

**Figure 7:**
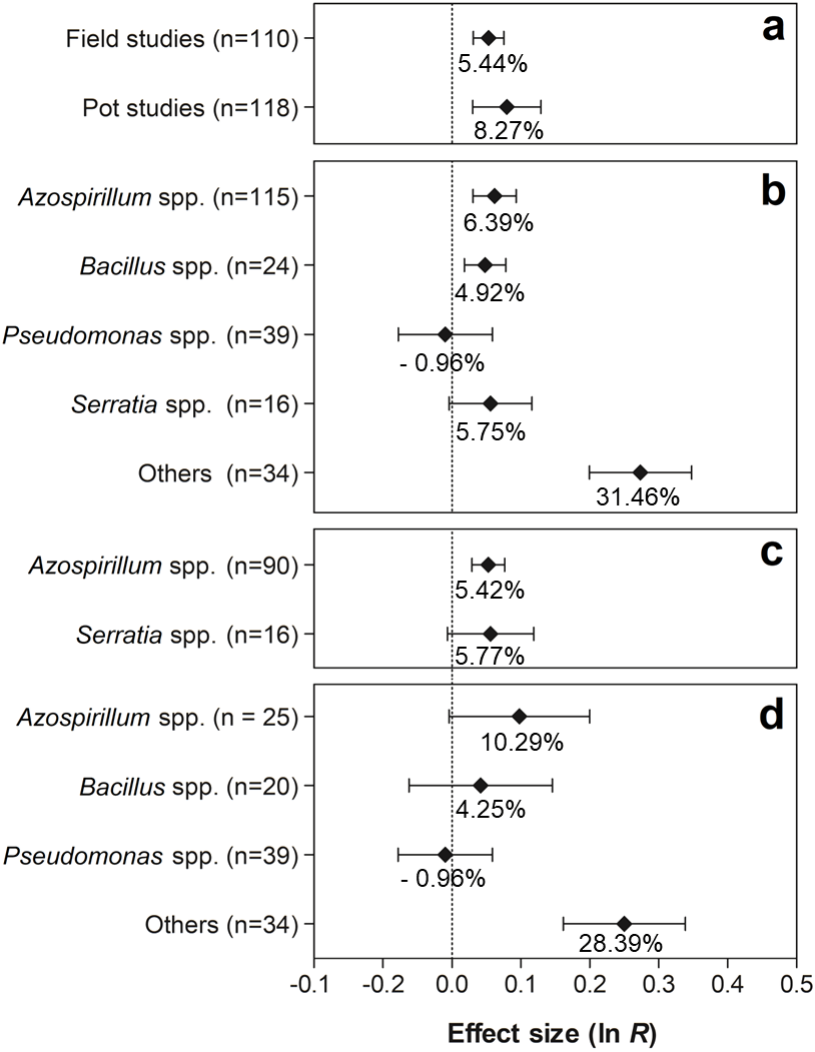
Effect sizes (ln *R*) of PGPR co-inoculation on shoot biomass grouped by the moderator variables: (A) experimental conditions; (B) genera of PGPR; (C) genera of PGPR under field conditions; and (D) genera of PGPR under pot conditions. The graph reflects the parameter estimates from the random-effects meta-analysis model and the error bars represent the 95% confidence interval. The values below the effect size of each variable are the percentages of the PGPR effect (ln *R* transformed back to the original values).

Figure 7 shows the effects of the moderator variables on the shoot biomass. When the experimental conditions were analyzed, it was possible to verify that the trials carried out under field and pot conditions presented significant values of 5.44% (95% CI [3.14–7.80%]) and 8.27% (95% CI [3.06–13.76%]), respectively (Fig. 7A). Both effect sizes can be considered similar, since the IC overlapped considerably. For this moderate variable, the genera *Azospirillum*, *Bacillus*, and others (*Actinomadura*, *Aeromonas*, *Enterobacter*, *Herbaspirillum*, *Methylobacterium*, *Nocardia*, *Nonomurae*, *Pseudocardia*, *Rhizobium*, *Stenotrophomonas*, and *Streptomyces*) were the only ones that presented positive effects on shoot biomass, leading to increases of 6.39% (95% CI [3.12–9.76%]), 4.92% (95% CI [1.82–8.12%]), and 31.46% (95% CI [22.07–41.58]), respectively (Fig. 7B). The partitioning of PGPR genera under field conditions indicated that co-inoculation with bacteria of the genus *Azospirillum* increased plant biomass in 5.42% (95% CI [2.95–7.95%]) (Fig. 7C). In the pot trials, an extra 28.39% (95% CI [17.50–40.27%]) in the average shoot biomass (Fig. 7D) was promoted by the grouped genera (*Actinomadura*, *Aerobonas, Enterobacter, Herbaspirillum, Methylobacterium, Nocardia, Nonomurae, Pseudocardia, Rhizobium, Stenotrophomonas*, and *Streptomyces*).

**Figure 8:**
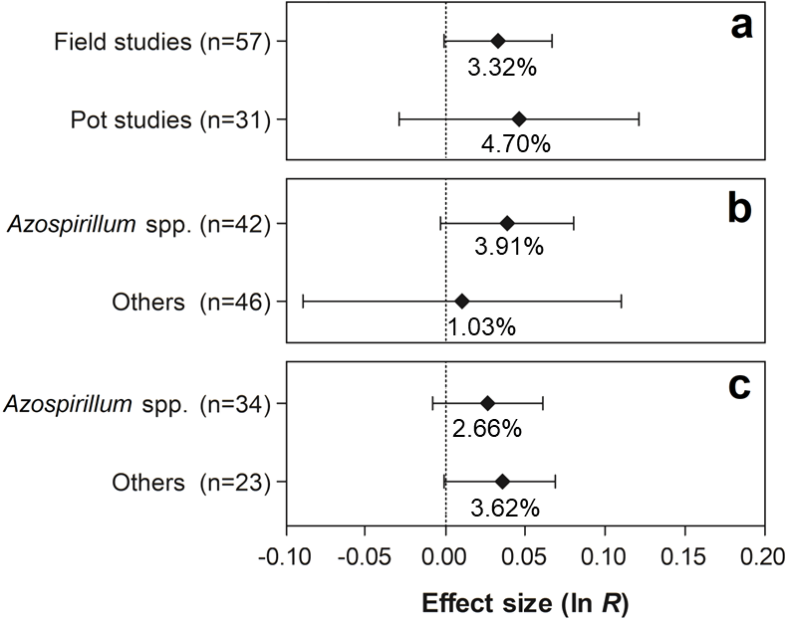
Effect sizes (ln *R*) of PGPR co-inoculation on the shoot N content grouped by the moderator variables: (A) experimental conditions; (B) genera of PGPR; and (C) genera of PGPR under field conditions. The graph reflects the parameter estimates from the random-effects meta-analysis model and the error bars represent the 95% confidence interval. The values below the effect size of each variable are the percentages of the PGPR effect (ln *R* transformed back to the original values).

**Figure 9:**
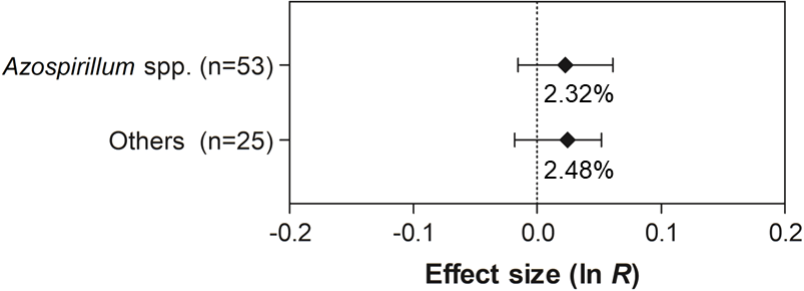
Effect sizes (ln *R*) of PGPR co-inoculation on grain yield considering the PGPR genera moderator variable. The graph reflects the parameter estimates from the random-effects meta-analysis model and the error bars represent the 95% confidence interval. The values below the effect size of each variable are the percentages of the PGPR effect (ln *R* transformed back to the original values).

For the traits shoot N content and grain yield, none of the differences were statistically significant, since the 95% CI of the moderator variables overlapped with zero (Figs. 8 and 9).

## Discussion

The soybean co-inoculation technology, in which traditional inoculation with selected strains of *Bradyrhizobium* is enhanced by the addition of bacteria considered plant growth promotors, has shown prominent results due to the complementary effects that these additional microorganisms promote. Whilst *Bradyrhizobium* acts as a microsymbiont, colonizing the plant root system and inducing the formation of nodules, PGPR increase root volume and number, thus enhancing the action of *Bradyrhizobium* in the supply of N biologically fixed to the plant, thereby potentially increasing grain yield (Hungria, Nogueira & Araujo, 2013; Hungria, Nogueira & Araujo, 2015). However, the literature lacks a quantitative synthesis of the real contribution of the co-inoculation technology to the soybean crop. Therefore, the results obtained in the present meta-analysis have great relevance for our understanding of the responses to the co-inoculation of symbiotic and associative bacteria in soybean cultivation, with implications for the commercialization of PGPR-based mixed inoculants.

Co-inoculation of soybean with PGPR provides increments in traits of great importance for obtaining high grain yields, such as number of nodules as well as nodule, root, and shoot biomass. Previous studies have demonstrated the existence of positive correlations between these traits and grain yield, although the interaction effects of genotype-genotype (macrosymbiont-microsymbiont) and genotype-environment are highlighted (Hwang et al., 2014; Cui et al., 2016; Thilakarathna & Raizada, 2017).

Meta-analysis studies quantifying the effects of PGPR on promoting plant-growth in different agricultural crops have been reported previously. Veresoglou & Menexes (2010) observed a significant increase of 23.81% in shoot biomass of wheat (*Triticum aestivum* L.) when inoculated with *Azospirillum* spp. Corroborating results were found by Rubin, Van Groenigen & Hungate (2017), who reported higher shoot and root biomass production (28 and 35%, respectively) induced by PGPR in a range of plant species. Furthermore, verifying the influence of inoculation with *Azospirillum* spp. in maize, interesting results were found by Zeffa et al. (2018), where the inoculated treatment out-yielded the control by 651 kg ha^−1^. In general, it is believed that the production of phytohormones by PGPR is one of the main mechanisms of action on the development of the host plant, whose effects are more prominent on the root system (Olanrewaju, Glick & Babalola, 2017; Puente et al., 2018). Interestingly, the symbiotic relationship between rhizobia and legumes is also mediated by bacterial phytohormones (Stacey et al., 1995; Imada et al., 2017). In this context, auxins produced by PGPR are believed to increase the number of root hairs, leading to the formation of rhizobia-soybean interaction sites (Schmidt, Messmer & Wilbois, 2015).

Puente et al. (2018) examined the effect of IAA on the co-inoculation response of soybean with *Bradyrhizobium* and *A. brasilense* and demonstrated that the increase in root system growth, which improves the soybean-*Bradyrhizobium* interaction, is a result of the action of phytohormones. Moreover, the authors co-inoculated soybean with *A. brasilense* Az39 (*ipdC+*) and with its respective mutant deficient in IAA biosynthesis (*ipdC-*). The authors observed that co-inoculation with *A. brasilense* Az39 promoted a greater efficiency in the *Bradyrhizobium-*soybean symbiosis when compared to the treatment of co-inoculation with the mutant (Az39 *ipdC-*) or the application of synthetic IAA and concluded that both the presence of *Azospirillum* and IAA biosynthesis by these bacteria are responsible for the positive effects of soybean co-inoculation with *Bradyrhizobium* and PGPR. Several other studies have linked phytohormone production to the successful interaction between rhizobia and legumes (Fukuhara et al., 1994; Srinivasan, Holl & Petersen, 1996; Vicario et al., 2015).

Although the correlation between nodulation parameters in soybean (nodule number and nodule biomass) is already widely described, the data assembled by the present meta-analysis indicated no significant increase in grain yield and shoot N content as a result of soybean co-inoculation compared to conventional inoculation (only *Bradyrhizobium*). It is important to emphasize that the meta-analysis for grain yield considered only data from field studies, in which the variables are difficult to control, such as the presence of native strains competing with the inoculant for nodulation. Furthermore, soybean responses to co-inoculation may vary according to plant genotype, bacterial strain, environmental conditions, as well as the quantity and quality of PGPR cells used as inoculants (Schmidt, Messmer & Wilbois, 2015; Pannecoucque et al., 2018; Chibeba et al., 2018). These variations in responses to co-inoculation were evident in the studies evaluated, which can be observed in the CI for different PGPR strains, in all the traits described.

The results of this meta-analysis point to a lack of a positive and significant contribution of co-inoculation to soybean grain yield. Nevertheless, indirect evidence indicates that the identification of inoculant strains that cause complementary effects on plant development is a crucial step for the development of more efficient soybean inoculants. Moreover, based on the analysis of the data gathered, it can be concluded that the improvement of soybean tolerance to abiotic stresses (such as drought and high temperatures) can be achieved by co-inoculation, since significant increases have been demonstrated for plant biomass and nodule number and biomass when this technique was applied.

In general, the results obtained in the present meta-analysis indicate the need for more experimental data from field experiments to produce more robust analyses to assess the real contribution of the co-inoculation technology for soybean cultivation. Among the traits that did not present statistical significance, shoot N content and grain yield were the ones with the lowest numbers of observations considered in the analysis. This situation is reinforced by the fact that co-inoculation of soybean with PGPR is more effective for experiments in pots compared to experiments conducted in the field. In addition to greater environmental control, the reader should bear in mind that experiments in pots present a less diverse native bacterial community compared to native soils, which means a greater competition between inoculant organisms and soil bacterial communities in field experiments (Çakmakçi et al., 2006).

## Conclusions

Our results demonstrated that the co-inoculation of soybean with *Bradyrhizobium* and other PGPR can substantially increase nodule number (11.40%), nodule biomass (6.47%), root biomass (12.84%), and shoot biomass (6.53%) in soybean. On the other hand, no significant differences were observed for shoot N content and grain yield. The bacterial genera *Azospirillum*, *Bacillus*, and *Pseudomonas* were more effective when compared to the genus *Serratia*. In general, co-inoculation results were more pronounced in experiments conducted in pots than in the field. The co-inoculation technology can be considered an economically viable and environmentally sustainable strategy for soybean cultivation.

##  Supplemental Information

10.7717/peerj.7905/supp-1 Supplemental Information 1 Meta-analysis rationale

10.7717/peerj.7905/supp-2 Table S1 PRISMA checklist

10.7717/peerj.7905/supp-3 Data S1 Raw data

## Additional Information and Declarations

### Data Availability

The following information was supplied regarding data availability: The raw measurements are available in the Supplemental Files.

## References

[ref-1] Ahemad M, Kibret M (2014). Mechanisms and applications of plant growth promoting rhizobacteria: current perspective. Journal of King Saud University—Science.

[ref-2] Atieno M, Herrmann L, Okalebo R, Lesueur D (2012). Efficiency of different formulations of *Bradyrhizobium japonicum* and effect of co-inoculation of *Bacillus subtilis* with two different strains of *Bradyrhizobium japonicum*. World Journal of Microbiology and Biotechnology.

[ref-3] Bai Y (2002). An inducible activator produced by a *Serratia proteamaculans* strain and its soybean growth-promoting activity under greenhouse conditions. Journal of Experimental Botany.

[ref-4] Çakmakçi R, Dönmez F, Aydın A, Şahin F (2006). Growth promotion of plants by plant growth-promoting rhizobacteria under greenhouse and two different field soil conditions. Soil Biology and Biochemistry.

[ref-5] Chang W-S, Lee H-I, Hungria M, Lugtenberg B (2015). Soybean production in the Americas. Principles of plant-microbe interactions.

[ref-6] Chibeba AM, Kyei-Boahen S, De Guimarães MF, Nogueira MA, Hungria M (2018). Feasibility of transference of inoculation-related technologies: a case study of evaluation of soybean rhizobial strains under the agro-climatic conditions of Brazil and Mozambique. Agriculture, Ecosystems & Environment.

[ref-7] Cochran WG (1954). The combination of estimates from different experiments. Biometrics.

[ref-8] Collino DJ, Salvagiotti F, Perticari A, Piccinetti C, Ovando G, Urquiaga S, Racca RW (2015). Biological nitrogen fixation in soybean in Argentina: relationships with crop, soil, and meteorological factors. Plant and Soil.

[ref-9] Cooper HM, Hedges LV, Valentine JC (2009). The handbook of research synthesis and meta-analysis.

[ref-10] Cui X, Dong Y, Gi P, Wang H, Xu K, Zhang Z (2016). Relationship between root vigour, photosynthesis and biomass in soybean cultivars during 87 years of genetic improvement in the northern China. Photosynthetica.

[ref-11] Delamuta JRM, Menna P, Ribeiro RA, Hungria M (2017). Phylogenies of symbiotic genes of *Bradyrhizobium* symbionts of legumes of economic and environmental importance in Brazil support the definition of the new symbiovars pachyrhizi and sojae. Systematic and Applied Microbiology.

[ref-12] Egamberdieva D, Jabborova D, Berg G (2016). Synergistic interactions between *Bradyrhizobium japonicum* and the endophyte *Stenotrophomonas rhizophila* and their effects on growth, and nodulation of soybean under salt stress. Plant and Soil.

[ref-13] Fukuhara H, Minakawa Y, Akao S, Minamisawa K (1994). The involvement of indole-3-acetic acid produced by *Bradyrhizobium elkanii* in nodule formation. Plant and Cell Physiology.

[ref-14] Gresshoff PM (2018). Molecular biology of symbiotic nitrogen fixation.

[ref-15] Hart C, Nguyen HT, Bhattacharyya MK (2017). The economic evolution of the soybean industry. The soybean genome.

[ref-16] Hedges LV, Gurevitch J, Curtis PS (1999). The meta-analysis of response ratios in experimental ecology. Ecology.

[ref-17] Higgins JPT, Thompson SG (2002). Quantifying heterogeneity in a meta-analysis. Statistics in Medicine.

[ref-18] Hungria M, Franchini JC, Campo RJ, Crispino CC, Moraes JZ, Sibaldelli RNR, Mendes IC, Arihara J (2006). Nitrogen nutrition of soybean in Brazil: contributions of biological N_2_ fixation and N fertilizer to grain yield. Canadian Journal of Plant Science.

[ref-19] Hungria M, Franchini JC, Campo RJ, Graham PH, Werner D, Newton WE (2005). The importance of nitrogen fixation to soybean cropping in South America. Nitrogen fixation in agriculture, forestry, ecology, and the environment.

[ref-20] Hungria M, Marco AN, Ricardo SA (2015). Alternative methods of soybean inoculation to overcome adverse conditions at sowing. African Journal of Agricultural Research.

[ref-21] Hungria M, Menna P, Delamuta JRM, De Bruijn FJ (2015). *Bradyrhizobium*, the ancestor of all rhizobia: phylogeny of housekeeping and nitrogen-fixation genes. Biological nitrogen fixation.

[ref-22] Hungria M, Nogueira MA, Araujo RS (2013). Co-inoculation of soybeans and common beans with rhizobia and azospirilla: strategies to improve sustainability. Biology and Fertility of Soils.

[ref-23] Hungria M, Nogueira MA, Araujo RS (2015). Soybean seed co-inoculation with *Bradyrhizobium* spp. and *Azospirillum brasilense*: a new biotechnological tool to improve yield and sustainability. American Journal of Plant Sciences.

[ref-24] Hwang S, Ray JD, Cregan PB, King CA, Davies MK, Purcell LC (2014). Genetics and mapping of quantitative traits for nodule number, weight, and size in soybean (*Glycine max* L.[Merr.]). Euphytica.

[ref-25] Imada EL, De Rolla dos Santos AAP, De Oliveira ALM, Hungria M, Rodrigues EP (2017). Indole-3-acetic acid production via the indole-3-pyruvate pathway by plant growth promoter *Rhizobium tropici* CIAT 899 is strongly inhibited by ammonium. Research in Microbiology.

[ref-26] Jordan DC (1982). Transfer of *Rhizobium japonicum* Buchanan 1980 to *Bradyrhizobium* gen. nov., a genus of slow-growing, root nodule bacteria from leguminous plants. International Journal of Systematic Bacteriology.

[ref-27] Liberati A, Altman DG, Tetzlaff J, Mulrow C, Gøtzsche PC, Ioannidis JP, Clarke M, Devereaux PJ, Kleijnen J, Moher D (2009). The PRISMA statement for reporting systematic reviews and meta-analyses of studies that evaluate health care interventions: explanation and elaboration. PLOS Medicine.

[ref-28] LPSN. List of Prokaryotic Names with Standing in Nomenclature (2019). http://www.bacterio.net/.

[ref-29] Mishra PK, Mishra S, Selvakumar G, Kundu S, Shankar Gupta H (2009). Enhanced soybean (*Glycine max* L.) plant growth and nodulation by *Bradyrhizobium japonicum* SB1 in presence of *Bacillus thuringiensis* KR1. Acta Agriculturae Scandinavica, Section B—Plant Soil Science.

[ref-30] Muthukumar T, Udaiyan K (2018). Coinoculation of bioinoculants improve *Acacia auriculiformis* seedling growth and quality in a tropical Alfisol soil. Journal of Forestry Research.

[ref-31] Nguyen H (2018). Achieving sustainable cultivation of soybeans Volume 1: breeding and cultivation techniques.

[ref-32] Olanrewaju OS, Glick BR, Babalola OO (2017). Mechanisms of action of plant growth promoting bacteria. World Journal of Microbiology and Biotechnology.

[ref-33] Oldroyd GED (2013). Speak, friend and enter: signalling systems that promote beneficial symbiotic associations in plants. Nature Reviews Microbiology.

[ref-34] Pan B, Vessey JK, Smith DL (2002). Response of field-grown soybean to co-inoculation with the plant growth promoting rhizobacteria *Serratia proteamaculans* or *Serratia liquefaciens*, and *Bradyrhizobium japonicum* pre-incubated with genistein. European Journal of Agronomy.

[ref-35] Pannecoucque J, Goormachtigh S, Ceusters J, Debode J, Van Waes C, Van Waes J (2018). Temperature as a key factor for successful inoculation of soybean with *Bradyrhizobium* spp. under cool growing conditions in Belgium. The Journal of Agricultural Science.

[ref-36] Pawar PU, Kumbhar CT, Patil VS, Khot GG (2018). Effect of co-inoculation of *Bradyrhizobium japonicum* and *Pseudomonas fluorescens* on growth, yield and nutrient uptake in soybean [*Glycine max* (L.) Merrill]. Crop Research.

[ref-37] Pérez JMM, Pascau J (2013). Image processing with imageJ: discover the incredible possibilities of ImageJ, from basic image processing to macro and plugin development.

[ref-38] Puente ML, Gualpa JL, Lopez GA, Molina RM, Carletti SM, Cassán FD (2018). The benefits of foliar inoculation with *Azospirillum brasilense* in soybean are explained by an auxin signaling model. Symbiosis.

[ref-39] Rubin RL, Van Groenigen KJ, Hungate BA (2017). Plant growth promoting rhizobacteria are more effective under drought: a meta-analysis. Plant and Soil.

[ref-40] Schmidt J, Messmer M, Wilbois K-P (2015). Beneficial microorganisms for soybean (*Glycine max* (L.) Merr), with a focus on low root-zone temperatures. Plant and Soil.

[ref-41] Schwarzer G (2007). meta: an R package for meta-analysis. R news.

[ref-42] Siqueira A, Ormeño Orrillo E, Souza R, Rodrigues E, Almeida LG, Barcellos F, Batista JS, Nakatani A, Martínez-Romero E, Vasconcelos AT, Hungria M (2014). Comparative genomics of *Bradyrhizobium japonicum* CPAC 15 and *Bradyrhizobium diazoefficiens* CPAC 7: elite model strains for understanding symbiotic performance with soybean. BMC Genomics.

[ref-43] Srinivasan M, Holl FB, Petersen DJ (1996). Influence of indoleacetic-acid-producing Bacillus isolates on the nodulation of *Phaseolus vulgaris* by *Rhizobium etli* under gnotobiotic conditions. Canadian Journal of Microbiology.

[ref-44] Stacey G, Sanjuan J, Luka S, Dockendorff T, Carlson RW (1995). Signal exchange in the *Bradyrhizobium*-soybean symbiosis. Soil Biology and Biochemistry.

[ref-45] Sugiyama A, Ueda Y, Takase H, Yazaki K (2015). Do soybeans select specific species of *Bradyrhizobium* during growth?. Communicative & Integrative Biology.

[ref-46] Thilakarathna MS, Raizada MN (2017). A meta-analysis of the effectiveness of diverse rhizobia inoculants on soybean traits under field conditions. Soil Biology and Biochemistry.

[ref-47] Tonelli ML, Magallanes-Noguera C, Fabra A (2017). Symbiotic performance and induction of systemic resistance against *Cercospora sojina* in soybean plants co-inoculated with *Bacillus* sp. CHEP5 and *Bradyrhizobium japonicum* E109. Archives of Microbiology.

[ref-48] Veresoglou SD, Menexes G (2010). Impact of inoculation with *Azospirillum* spp. on growth properties and seed yield of wheat: a meta-analysis of studies in the ISI Web of Science from 1981 to 2008. Plant and Soil.

[ref-49] Vicario JC, Gallarato LA, Paulucci NS, Perrig DS, MÁ Bueno, Dardanelli MS, Cassán FD, Okon Y, Creus CM (2015). Co-inoculation of Legumes with *Azospirillum* and Symbiotic Rhizobia. Handbook for azospirillum.

[ref-50] Viechtbauer W (2010). Conducting meta-analyses in R with the metafor package. Journal of Statistical Software.

[ref-51] Wickham H (2016). ggplot2.

[ref-52] Yadav MR, Kumar R, Parihar CM, Yadav RK, Jat SL, Ram H, Meena RK, Singh MB, Verma AP, Ghoshand A, Jat ML (2017). Strategies for improving nitrogen use efficiency: a review. Agricultural Reviews.

[ref-53] Yan T, Zhu J, Yang K (2018). Leaf nitrogen and phosphorus resorption of woody species in response to climatic conditions and soil nutrients: a meta-analysis. Journal of Forestry Research.

[ref-54] Zeffa DM, Fantin LH, Dos Santos OJAP, De Oliveira ALM, Canteri MG, Scapim CA, Gonçalves LSA (2018). The influence of topdressing nitrogen on *Azospirillum* spp. inoculation in maize crops through meta-analysis. Bragantia.

[ref-55] Zuffo AM, De Rezende PM, Bruzi AT, Ribeiro ABM, Zambiazzi EV, Soares IO, Vilela NJD, Bianchi MC (2016). Soybean cultivars agronomic performance and yield according to doses of *Azospirillum brasilense* applied to leaves. Australian Journal of Crop Science.

