# Supplementary material for: Effects of plant growth-promoting rhizobacteria on co-inoculation with Bradyrhizobium in soybean crop: a meta-analysis of studies from 1987 to 2018
Source: PeerJ. 2020 Jan 9;8:e7905. doi: 10.7717/peerj.7905 (PMC6955106; doi:10.7717/peerj.7905)
Supplement: Supplemental Information 1 [file peerj-08-7905-s001.docx]

September, 02 - 2019

Ana Ribeiro Barros

Academic Editor PeerJ

Dear Editor,

The co-inoculation of soybean with *Bradyrhizobium* and other plant growth-promoting rhizobacteria (PGPR) is considered a promising technology. Although it is considered a promising technology, the co-inoculation of soybean has shown contrasting results. Therefore, what is not yet clear is the impact of co-inoculation on soybean grain yield. In view of this, the statistical technique known as meta-analysis may be a powerful tool to determine the real effects of the co-inoculation of PGPR and *Bradyrhizobium* on soybean cultivation. Therefore, the aim of this study was to investigate and solve the inconsistency of results using a meta-analysis. A total of 42 published articles were examined, all of which considered the effects of co-inoculation of PGPR and *Bradyrhizobium* on the number of nodules, nodule biomass, root biomass, shoot biomass, shoot nitrogen content, and grain yield of soybean. We also determined whether the genus of the PGPR used as co-inoculant, as well as the experimental conditions, altered the effect size of the PGPR.

Best regards,

Leandro SA Gonçalves

Articles:

Rubin RL, van Groenigen KJ, Hungate BA. 2017. Plant growth promoting rhizobacteria are more effective under drought: a meta-analysis. *Plant and Soil* 416: 309-323.

Veresoglou SD, Menexes G. 2010. Impact of inoculation with *Azospirillum* spp. on growth properties and seed yield of wheat: a meta-analysis of studies in the ISI Web of Science from 1981 to 2008. *Plant and Soil* 337:469–480.

Zeffa DM, Fantin LH, Santos OJAP dos, Oliveira ALM de, Canteri MG, Scapim CA, Gonçalves LSA. 2018. The influence of topdressing nitrogen on *Azospirillum* spp. inoculation in maize crops through meta-analysis. *Bragantia* 77:493–500.
